# Characteristics of Resting-State Functional Connectivity in HIV-Associated Neurocognitive Disorder

**DOI:** 10.1371/journal.pone.0153493

**Published:** 2016-04-22

**Authors:** Hea Won Ann, Suhnyoung Jun, Na-Young Shin, Sanghoon Han, Jin Young Ahn, Mi Young Ahn, Yong Duk Jeon, In Young Jung, Moo Hyun Kim, Woo Yong Jeong, Nam Su Ku, June Myung Kim, Davey M. Smith, Jun Yong Choi

**Affiliations:** 1 Department of Internal Medicine, Yonsei University College of Medicine, Seoul, South Korea; 2 Department of AIDS Research Institute, Yonsei University College of Medicine, Seoul, South Korea; 3 Department of Psychology, Yonsei University, Seoul, South Korea; 4 Department of Radiology, Ewha Womans University School of Medicine, Seoul, South Korea; 5 Department of Medicine, University of California San Diego, La Jolla, CA, United States of America; 6 Veterans Affairs San Diego Healthcare System, San Diego, CA, United States of America; University of Rome, ITALY

## Abstract

**Background:**

HIV-associated neurocognitive disorder (HAND) can occur in patients without prior AIDS defining illness and can be debilitating. This study aimed to evaluate the difference in the patterns of intrinsic brain activity between patients with or without HAND for deepening our understanding of HAND.

**Methods:**

We evaluated 24 HIV-infected individuals, 12 with previously diagnosed HAND and 12 previously diagnosed without HAND, and 11 seronegative individuals. These individuals then underwent repeat NP testing and a functional brain MRI scan. For functional MRI analysis, seed-based analysis with bilateral precuneus cortex seed was applied.

**Results:**

Among the 12 individuals with previously diagnosed HAND, 3 showed improvement of their neurocognitive function and 1 was excluded for worsening liver disease. Among the 12 patients who previously had normal neurocognitive function, 2 showed neurocognitive impairment. Overall, the HAND group, who had impaired cognitive function at the time of MRI scan, showed significant decrease of resting status functional connectivity between bilateral precuneus and prefrontal cortex (PFC) compared with nonHAND group, those who had normal neurocognitive function (Corrected *P*<0.05). The functional connectivity with the right inferior frontal operculum and right superior frontal gyrus was positively correlated with memory and learning ability.

**Conclusions:**

This cross-sectional study found a significant difference in fMRI patterns between patients with and without HAND. Decreased functional connectivity between precuneus and PFC could be possible functional substrate for cognitive dysfunction in HIV patients, which should be characterized in a longitudinal study.

## Introduction

HIV associated neurocognitive disorder (HAND) is a general term that incorporates a number of neurological impairments with a range of severity including asymptomatic neurocognitive impairment (ANI), mild neurocognitive disorder (MND), and HIV associated dementia (HAD) based on self-report about interference with daily functioning [1]. Impairments in daily functioning associated with HAND include employment, taking medications, financial management, cooking etc [2, 3]. It is a quite common medical condition even during effective antiretroviral therapy (ART) with the prevalence in HIV-infected individuals between 16–52% [1, 4, 5]. Elaborate neuropsychological (NP) examinations are required to diagnose HAND [1], and there is no consensus on how to detect HAND via imaging tools.

Recent developments in functional magnetic resonance imaging (fMRI) make it an attractive option is getting help in understanding pathophysiology of HAND, since it can evaluate connectivity and communications between brain regions [6]. The dysfunctions in these communications between brain regions are likely associated with the mechanisms underlying HAND pathogenesis [7]. In this study, we evaluated whether brain fMRI can identify a pattern associated with HAND.

## Methods

### Study design

Twelve male patients who diagnosed with HAND in 2011 [5] and 12 male patients without HAND (also confirmed in 2011, same test procedure with HAND patients) were randomly recruited from HIV cohort. As a control group, 11 age- and sex- matched seronegative subjects were also recruited. Potential participants were not considered for enrollment if they had: (1) recent and/or significant traumatic brain injury, (2) a neurological disorder not related to HIV infection, (3) infections that can affect the CNS, (4) a significant CNS opportunistic infection based upon history and/or neuromedical examination (5) a current or past psychotic disorder, (6) significant substance use (more than three alcoholic drinks per day over the last month or recreational drug use more than once per week during the last month), (7) symptoms of a current, active infection, a body temperature of >38.5°C at the time of recruitment or current treatment for a serious, systemic infection within 3 months, (8) colour blindness, (9) a hearing deficit that appears to affect auditory comprehension [1]. All subjects provided informed consent and received standardized neurological, NP, and functional assessments at enrollment. This study was approved by the Institutional Review Board of the hospital (IRB #4-2013-0499).

### Demographic and clinical data

Age, sex, duration of education, performance status [8], body mass index, hemoglobin level, estimated glomerular filtration ratio, antiretroviral regimen, CNS penetration effectiveness(CPE) [9], initial CD4 T-cell count, pre-cART CD4 T-cell count, current CD4 T-cell count, lowest CD4 T-cell count, initial viral load (VL), pre-cART VL and current VL and highest VL were available and included in the analysis.

### Neuropsychological tests

The NP exam and fMRI scans were carried out within a month of study enrollment for each participant. The neurocognitive status assessed six ability domains. (1) speed of information processing using the Korean version of Wechsler Adult Intelligence Scale(K-WAIS) digit symbol subtest, trail making test part A; (2) memory (learning and recall) using the Korean version of auditory verbal learning test and complex figure test; (3) abstraction/executive function using the Wisconsin Card Sorting Test, K-WAIS similarity subtest and trail making test part B; (4) attention/working memory using the K-WAIS digit span subtest; (5) sensory perception/motor skills using the Grooved Pegboard Test; (6) verbal/language using the K-WAIS vocabulary subtest. The decision of functional impairment was made when the score of a patient show below 1 standard deviation (SD) of demographically corrected normative means.

To evaluate functional status, eight questions designed by Ku *et al*. [5] (based on the suggestions of Antinori *et al*. [1]) were used. The questions can be translated as below: ‘Is it hard to take medication in the correct dosages at the correct time?’; ‘Is it hard to manage financial matters independently (budgeting, writing cheques, paying rent and bills, going to the bank)?’; ‘Is it hard to perform household tasks alone or with occasional assistance?’; ‘Do you have trouble managing your daily schedule?’; ‘Do you make more mistakes in your working?’; ‘Do you need more time than before to do the same amount of work?’; ‘Do you find it more difficult than before to carry out tasks successfully?’; ‘Are you less able to produce your best work?’.

The Frascati criteria [1] were used to diagnose HAND, and HAND was sorted to asymptomatic neurocognitive impairment (ANI), mild neurocognitive disorder (MND) and HIV-associated dementia (HAD).[1]

### Functional MRI data acquisition

All participants took a resting-state fMRI on a 3T MR scanner (MAGNETOM Tim Trio, Siemens Healthcare, Erlangen, Germany) and functional data was acquired using a gradient-echo planar pulse sequence. For each subject, 150 axial volume scans were obtained with the following parameters: TR = 3000 msec, TE = 30 msec, FOV = 192 x 192 mm2, voxel size = 3 x 3 x 3 mm3, slice number = 50 (interleaved). In order to securely support the head and minimize head movement, vacuum molded cushions and soft pads were used. Total scan time was 7 min 39 sec.

### Functional MRI data analyses

All functional images underwent preprocessing using Statistical parametric mapping software (SPM8, Wellcome Department of Imaging Neuro-Science, London, U.K.) and the SPM8-based pipeline implemented in the Data Processing Assistant for Resting-State fMRI (DPARSF, version 2.2) [10]. The first 4 volumes were not analyzed to allow magnetization stabilization. The remaining 146 volumes were corrected by resampling all slices relative to the middle slice in temporal order. The functional images were realigned and spatially normalized to the Montreal Neurological Institute (MNI) template provided with SPM8. Images were resampled into 3-mm cubes, followed by spatial smoothing with a 8-mm full-width, half-maximum (FWHM) isotropic Gaussian kernel. REST implemented in the DPARSF was used for linear regression analysis to remove possible spurious variables: six parameters obtained by rigid body correction of head motion, mean signals from cerebrospinal fluid, averaged white matter signals, and averaged signals from whole brain. Unless stated otherwise, all statistical analyses were corrected based on Monte Carlo simulation that helps determine the cluster extent threshold in a specific *p* value. After 10,000 iterations, the probability of each cluster extent was calculated and the cluster extent that yielded similar to *P* < 0.05 (i.e., 15 contiguous resampled voxels) was selected for use in voxel extent thresholding with an individual voxel threshold of *P* = 0.005 [11].

To perform functional connectivity (FC) analysis, the average blood-oxygen level dependent (BOLD) time series (TS) from the regions-of-interest (ROIs) in the left and right precuneus were calculated. The ROIs comprised the bilateral precuneus, as such regions are considered to be a structural and functional hub in the brain connectome.[12, 13] FC between BOLD TS of all voxels and the BOLD TS of ROIs were calculated and generated voxel-wise partial correlation coefficients (GCs) to create FC pattern maps.

### Statistical analyses

An independent t-test or χ2 test was used to assess differences in each variable between participants with and without HAND. Two-sampled t-tests were performed to find differences in FC patterns between the groups as a second-level random-effects analysis, and two factors (i.e., age and sex) were used as covariates. To define FC related with behavioral performance, we performed correlation analysis with FC and NP test results. The 3-by-3-by-3 cube masks centering the peak coordinates which showed significantly decreased FC with precuneus seed in HAND and nonHAND were defined as ROIs. The mean correlation value of each mask was calculated for each HIV subject. Correlation analyses compared the list of mean correlation values with the NP test scores examining significantly impaired cognitive domains in HAND compared to nonHAND.

## Results

### Characteristics of study participants

Among the 12 participants who were diagnosed with HAND previously [5], 3 had improvement of their neurocognitive function and 8 continued to demonstrate impaired neurocognitive function at the time of enrollment. One individual was excluded because of his liver cirrhosis progression. Among the 12 participants who showed normal neurocognitive function previously [5], 10 remained normal and 2 showed worsened neurocognitive function. Therefore, at the time of this study, 10 participants had impaired neurocognitive function (HAND group) and 13 had normal neurocognitive function (nonHAND group). In HAND group, 2 had asymptomatic neurocognitive impairment and 8 had mild neurocognitive impairment. No participants had HAD. All of the participants, including seronegative control group, were male. There were no differences in age and years of education between the three groups. There were also no differences in an CD4 count and CPE measures, and ART regimens between the HAND and nonHAND groups, but the HAND group did have a higher peak HIV RNA levels and HIV RNA levels at enrollment than the nonHAND group (p<0.05) (Table 1). Irrespective of group, functional impairment was demonstrated mostly in abstraction/executive (n = 9) and sensory perception/motor skills (n = 10) domains. All of the participants in seronegative control group showed normal cognitive function (Table 2).

**Table 1:** Baseline characteristics of study participants.

	HAND (n = 10)	nonHAND (n = 13)	Control (n = 11)	p-value
Sex(male)	100%	100%	100%	
Age(years)	56.0±8.2	52.8±6.2	54.7±4.2	0.47
BMI(kg/m2)	23.6±2.0	22.3±2.4	25.2±2.0	0.018
education (years)	13.1±4.4	12.7±3.3	12.7±1.6	0.949
Hemoglobin	14.2±1.5	14.0±1.4	14.3±2.2	0.931
eGFR	80.7±15.3	82.5±10.6	85.6±7.5	0.585
CD4 at diagnose (cells/uL, mean±SD)	203.8±220.7	284.1±239.1		0.441
VL at diagnose (log10 copies/mL) (mean ± SD)	5.5±5.5	4.3±4.3		0.019
pre ART CD4 (cells/uL, mean±SD)	201.8±151.9	224.5±91.5		0.722
Pre ART VL (log10 copies/mL) (mean ± SD)	5.2±5.5	4.8±4.9		0.304
current CD4 (cells/uL, mean±SD)	525.8±252.2	697.9±194.2		0.078
current VL (log10 copies/mL) (mean ± SD)	1.4±1.0	1.3±0.0		0.264
Nadir CD4 (cells/uL, mean±SD)	188.9±183.1	216.8±147.9		0.69
Highest VL (log10 copies/mL) (mean ± SD)	5.4±5.4	4.8±4.9		0.034
ART regimen (n, %)				
no treatment	0	0		
2NRTIs+PI	6(60)	5(38)		0.327
2NRTIs+NNRTI	3(30)	4(31)		0.97
2NRTIs+II	1(10)	4(31)		0.251
CPE score(mean±SD)	7.60±1.35	7.92±0.76		0.474
Change in ART	5(50)	5(38)		0.6

Initial: at diagnose.

**Table 2:** Impairment of domains of neurocognitive function.

Domain		Patients who showed impairment [n, (%)]			
	HAND (n = 10)		nonHAND (n = 13)	Seronegative Control (n = 11)	p-value
Verbal/language	1 (10)		0 (0)	0 (0)	0.343
Speed of information processing	0 (0)		0 (0)	0 (0)	-
Memory (learning and recall)	5 (50)		0 (0)	0 (0)	0.015
Abstraction/executive	8 (80)		1 (7.7)	0 (0)	<0.001
attention/working memory	0 (0)		0 (0)	0 (0)	-
Sensory perceptual/motor skills	8 (80)		2 (15.4)	0 (0)	0.001

### Brain fMRI

**Fig 1:**
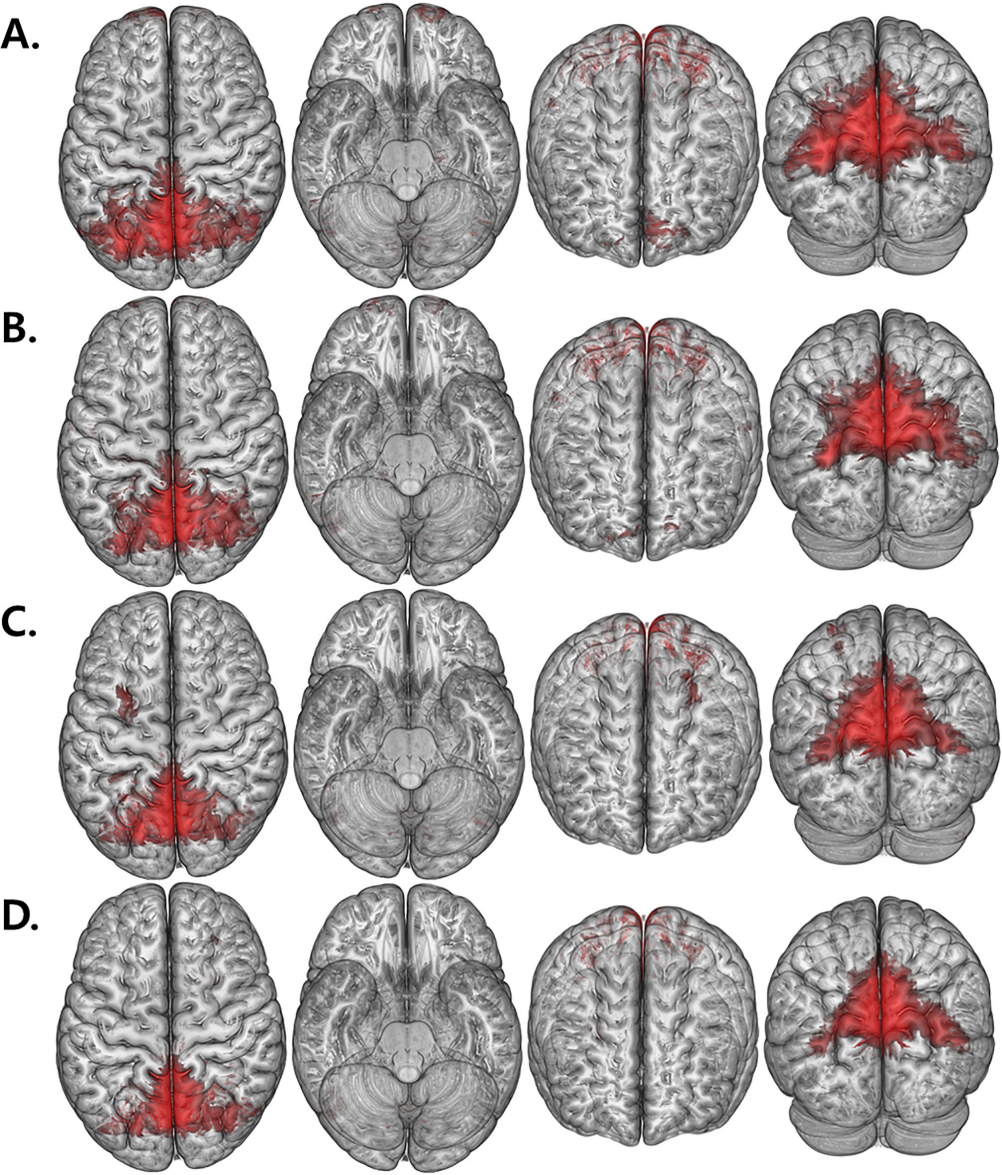
Renderd *t* maps of FC value. (A, B) show the FC maps of HAND with the seed located in the left and right precuneus, respectively. (C, D) show the FC maps of nonHAND group with the seed located in the left and right precuneus (P < .005, uncorrected).

**Fig 2:**
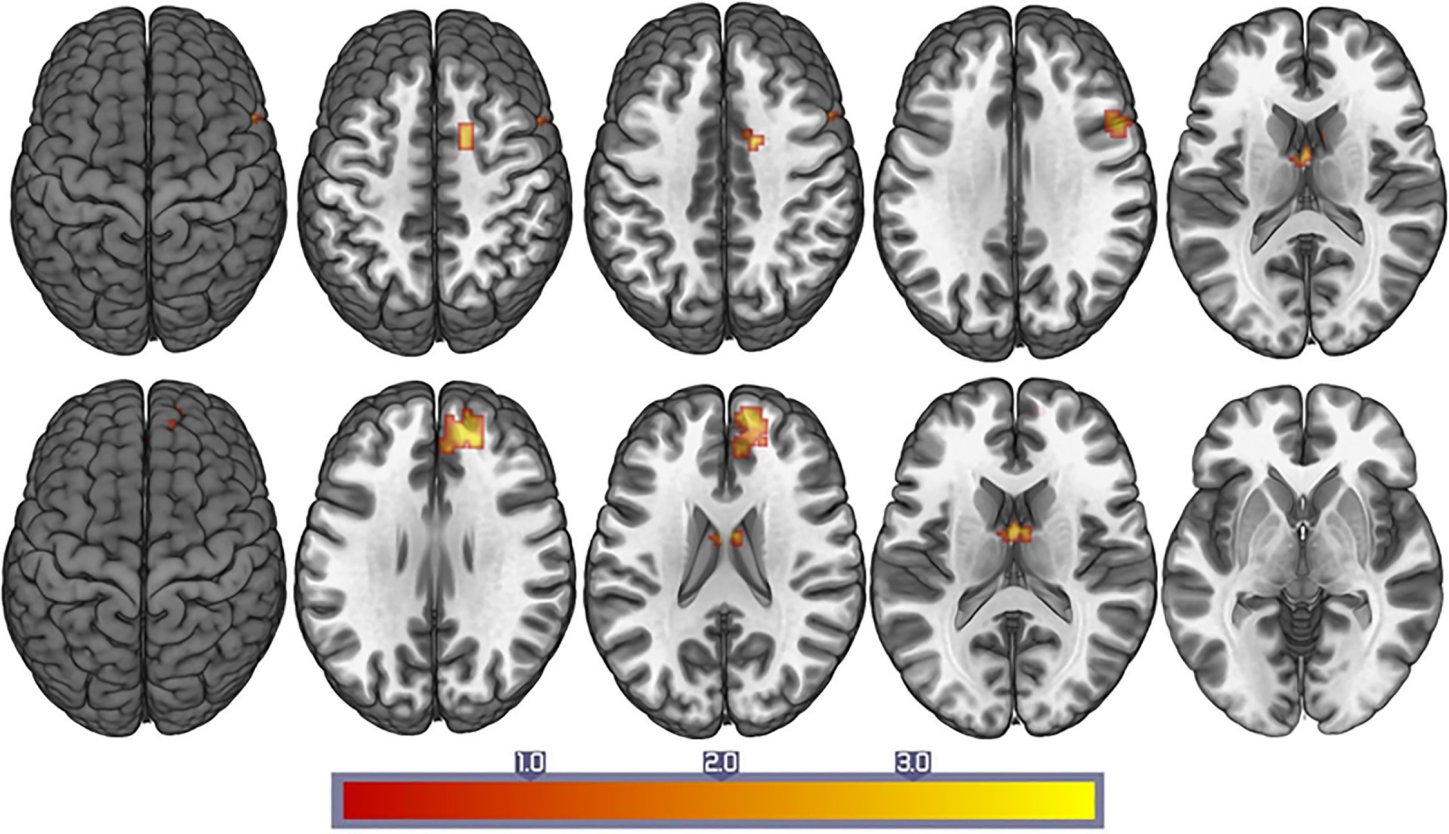
The differences of resting connectivity between HAND group and nonHAND group. Upper images display the regions showing the FC with the left precuneus (P < .05, corrected). Lower images display the regions showing the FC with the right precuneus (P < .05, corrected).

The intragroup FC patterns of each ROI were revealed using one-sample *t*-tests (Fig 1). In comparison with HAND and nonHAND groups, we found a decreased resting state FC between both precuneus with prefrontal cortex (PFC) regions (Corrected *P*<0.05; Fig 2 and Table 3). Interestingly, when comparing the nonHAND and seronegative group, bilateral frontal areas, temporal and cerebellum also showed significantly decreased FC (Table 4).

**Table 3:** Decreased resting state functional connectivity in HAND group compared to nonHAND group.

	Seed	Connected regions	Side	MNI coordinates			Maximum *t*	Mean *r* value		*P* value
				x	y	z		nonHAND	HAND	
HAND < nonHAND	Left Precuneus cortex	Inferior Frontal Operculum	R	51	9	27	3.58	0.028	-0.217	*<* .001
	Right Precuneus cortex	Superior Frontal Gyrus	R	18	45	27	3.93	-0.045	-0.211	*<* .001
HAND > nonHAND	Left Precuneus	Superior Temporal Pole	R	60	9	-6	5.39	-0.32	-0.006	*<* .001
		Cerebellum	R	42	-36	-30	4.09	-0.097	0.129	*<* .001
	Right Precuneus	Superior Temporal Pole	R	60	9	-6	4.66	-0.219	0.041	*<* .001
		Superior Temporal Gyrus	L	-51	0	-6	4.27	-0.222	0.013	*<* .001
		Lingual Gyrus	L	-15	-87	-15	3.98	-0.132	0.114	*<* .001
		Superior Orbitofrontal Gyrus	L	-21	48	-18	3.47	-0.13	0.09	*<* .001
		Superior Temporal Gyrus	R	69	-27	12	3.16	-0.113	0.118	*<* .001

**Table 4:** Decreased resting state functional connectivity in nonHAND group compared to seronegative control.

Seed	Connected regions	Side	MNI coordinates			Maximum *t*	Mean r value		*P* value
			x	y	z		nonHAND	seronegative control	
Left Precuneus	Superior Medial Frontal	R	3	63	30	5.27	-0.102	0.033	<0.001
	Angular	R	57	-57	36	4.81	-0.068	0.091	<0.001
	Hippocampus	R	27	-18	-15	4.48	-0.193	0.024	<0.001
	Middle Orbitofrontal	L	-6	63	-9	4.24	0.043	0.117	<0.001
	Precuneus	R	15	-60	30	4.07	0.428	0.485	<0.001
	Middle Temporal	R	63	-15	-15	3.84	-0.171	0.004	<0.001
	Superior Orbitofrontal	R	24	63	0	3.67	-0.065	0.019	<0.005
	Cerebellum	L	-48	-57	-39	3.53	-0.028	-0.036	<0.001
Right Precuneus	Hippocampus	R	27	-18	-18	4.14	-0.157	0.054	<0.001
	Precuneus	R	6	-48	12	4.08	0.398	0.492	<0.001
	Angular	R	57	-57	36	3.91	-0.001	0.063	<0.001
	Middle Temporal	R	69	-12	-9	3.78	-0.198	-0.042	<0.001
	Middle Temporal	L	-51	-21	-12	3.76	-0.174	-0.134	<0.001
	Precuneus	R	12	-60	27	3.49	0.547	0.591	<0.005
	Middle Orbitofrontal	R	6	60	-6	3.46	-0.014	0.089	<0.005

### Correlation Analyses between FC and NP test results

The right inferior frontal operculum and right superior frontal gyrus which had significantly decreased FC with precuneus in HAND compared to nonHAND were selected as ROIs for the correlation analysis. Among the NP tests examining memory, abstraction/executive, and sensory perceptual/motor skills domains which were significantly impaired in HAND relative to nonHAND, only tests examining memory domain were positively correlated with FC between precuneus and above-mentioned ROIs: both higher FC between left precuneus and right inferior frontal operculum (*r* = .46, *P* = .025) and right precuneus and right superior frontal gyrus (*r* = .59, *P* = .003) were correlated with higher score in K-AVLT delayed recall test; higher FC between right precuneus and right superior frontal gyrus were correlated with higher score in K-AVLT delayed recognition test (*r* = .56, *P* = .006; Table 5).

**Table 5:** Correlation analyses of decreased functional connectivity and neuropsychological function.

Regions of Interests [x y z]	Seed	NP tests					
		NP1	NP2	NP3	NP4	NP5	NP6
R Inferior Frontal Operculum [51 9 27]	L precuneus	0.38	0.46*	0.25	0.23	0.25	0.28
	R precuneus	0.46*	0.63**	0.59**	0.12	0.10	0.19
R Superior Frontal Gyrus [18 45 27]	L precuneus	0.34	0.40	0.17	0.22	0.15	0.16
	R precuneus	0.36	0.59**	0.56**	0.25	0.19	0.28

Note. Data are all Pearson’s *r* values. Values in the brackets are MNI coordinates. NP1: K-AVLT Total (Trials 1–5); NP2: K-AVLT Delayed Recall; NP3: K-AVLT Delayed Recognition; NP4: KCFT Copy; NP5: KCFT Immediate Recall; NP6: KCFT Delayed Recall.

* *P* < 0.05.

** Bonferroni-corrected *P* < 0.05.

## Discussion

This study was designed to see the difference of FC in fMRI between HAND and nonHAND patients. To tackle this, we re-evaluated 24 HIV-infected individuals after 2.5 years, and found that 8.3% of these individuals demonstrated neurocognitive decline, 75% were stable, and 12.5% improved. Sensitivity, specificity, and inherent variability (in-patient variability) of NP testing need to be taken into account when interpretating HAND diagnosis and classification, especially in the setting of our small sample sizes. However, these observed changes are consistent with a previous report by Heaton et al. that showed 23% of patients had their cognitive function decline, 61% remained stable, and 17% showed improved cognitive function [14].

Overall, this study found differences in fMRI patterns between HIV-infected with and without HAND, and also between nonHAND and seronegative individuals. In particular, these results suggest that a pattern on fMRI can be used to distinguish HIV-infected people with and without HAND, mostly associated with differences in FC in left and right precuneus and prefrontal brain regions.

Previous imaging studies have found that cerebral atrophy, decrease of baseline cerebral blood flow and cerebral signal intensity abnormalities are associated with HAND [15–17]. Further, Ances et al. reported decreased functional BOLD response of HIV–infected individuals compared with HIV-uninfected controls [18], and Küper et al. reported decrease of gray matter in the anterior cingulate and temporal cortices, along with longer duration of HIV infection or worse cognitive function [19]. Plessis et al. found that fronto-striatal dysfunction of HIV patients was associated with cognitive impairment by fMRI [20]. In our study, we found that there were significant FC differences between the nonHAND group patients and seronegative controls around that frontal and temporal areas, and this is concordant with several previous studies [16, 17, 19, 20].

On correlation analysis, differences in FC in the frontal areas between HAND and nonHAND groups were related to performance capability. Among the brain regions that showed significant differences in the FC comparison between HAND and nonHAND, we selected the right inferior frontal operculum and right superior frontal gyrus as the ROIs of the correlation analysis. FC of the ROIs was positively correlated with scores from the NP test for memory, learning, and recall. Moreover, FC between precuneus and frontal areas were significantly correlated with verbal memory/learning ability, keeping in line with previous studies [21]. Therefore, we speculated the altered resting-state FC within these areas might have critical role in cognitive dysfunction in HIV-infected patients.

This study has several limitations. First, while this study showed differences in fMRI between groups, this is an association and not a causation, and since this is a cross-sectional study, then prognostic and diagnostic validations cannot be determined. Second, the sample size was too small to analyze the risk factors of fluctuation in cognitive function. If we had follow up result of NP test of seronegative controls, we could adjust cognitive decline according to aging. Third, all the patients with HAND had asymptomatic or mild neurocognitive dysfunction, so we could not assess the individuals with severe cognitive impairment, like HIV-associated dementia. Further, the seronegative control group showed normal results in all 6 functional domains, which could bias the fMRI differences between groups. Lastly, all the participants were male, so these results are likely not generalizable to women.

In conclusion, there was significant difference between HAND and nonHAND groups on resting state FC in functional brain MRI. There was also significant difference between the nonHAND group and seronegative control group. Future studies will need evaluate these fMRI patterns prospectively in the diagnosis of people who will ultimately develop HAND, and if functional brain MRI may be useful to screen for HAND.

## Supporting Information

S1 Dataset Variables and data for analyses. (XLSX)
